# Supplementary material for: Silver Doped Magnesium Ferrite Nanoparticles: Physico-Chemical Characterization and Antibacterial Activity
Source: Materials (Basel). 2021 May 26;14(11):2859. doi: 10.3390/ma14112859 (PMC8198861; doi:10.3390/ma14112859)
Supplement: Supplementary file 1 [file materials-14-02859-s001.zip › materials-1233854-supplementary.pdf]

materials

Supplementary Materials

Silver Doped Magnesium Ferrite Nanoparticles: Physico-Chemical Characterization and Antibacterial Activity

Erika Fantozzi [1], Erlinda Rama [2], Cinzia Calvio [2], Benedetta Albini [3], Pietro Galinetto [3] and Marcella Bini [1,*]

[1] Department of Chemistry, University of Pavia, viale Taramelli 16, 27100 Pavia, Italy; erika.fantozzi01@universitadipavia.it
[2] Department of Biology and Biotechnology "L. Spallanzani", University of Pavia, via Ferrata 9, 27100 Pavia, Italy; erlinda.rama01@universitadipavia.it (E.R.); cinzia.calvio@unipv.it (C.C.)
[3] Department of Physics and CNISM, University of Pavia, via Bassi 6, 27100 Pavia, Italy; benedetta.albini01@universitadipavia.it (B.A.); pietro.galinetto@unipv.it (P.G.)
* Correspondence: bini@unipv.it

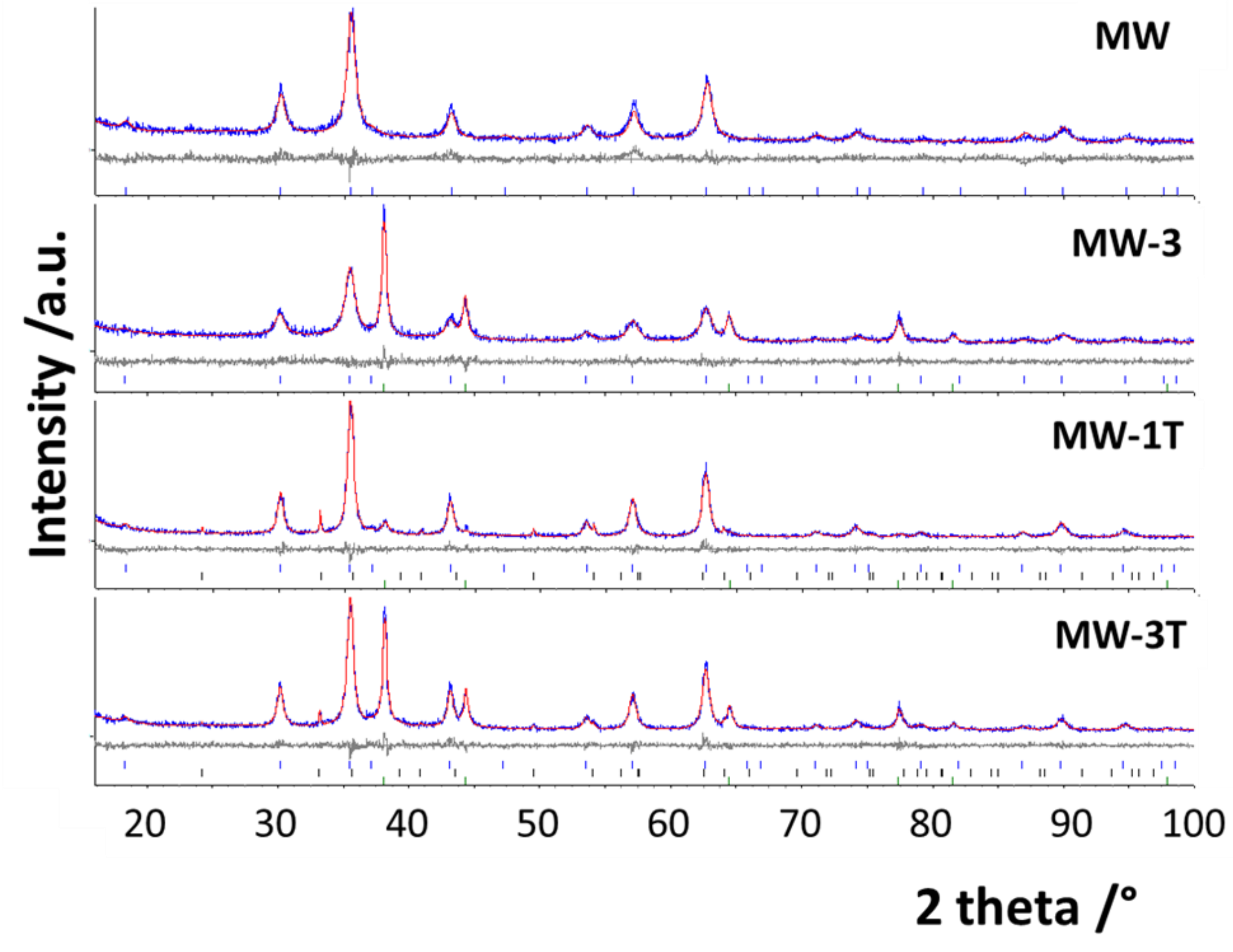

Figure S1. Rietveld refinements of the XRPD patterns of the MW samples indicated above each graph. The experimental curve (blue) is compared with the calculated one (red). On the bottom, the difference curve (grey) and the bars at the expected positions of the reflections for the different phases.

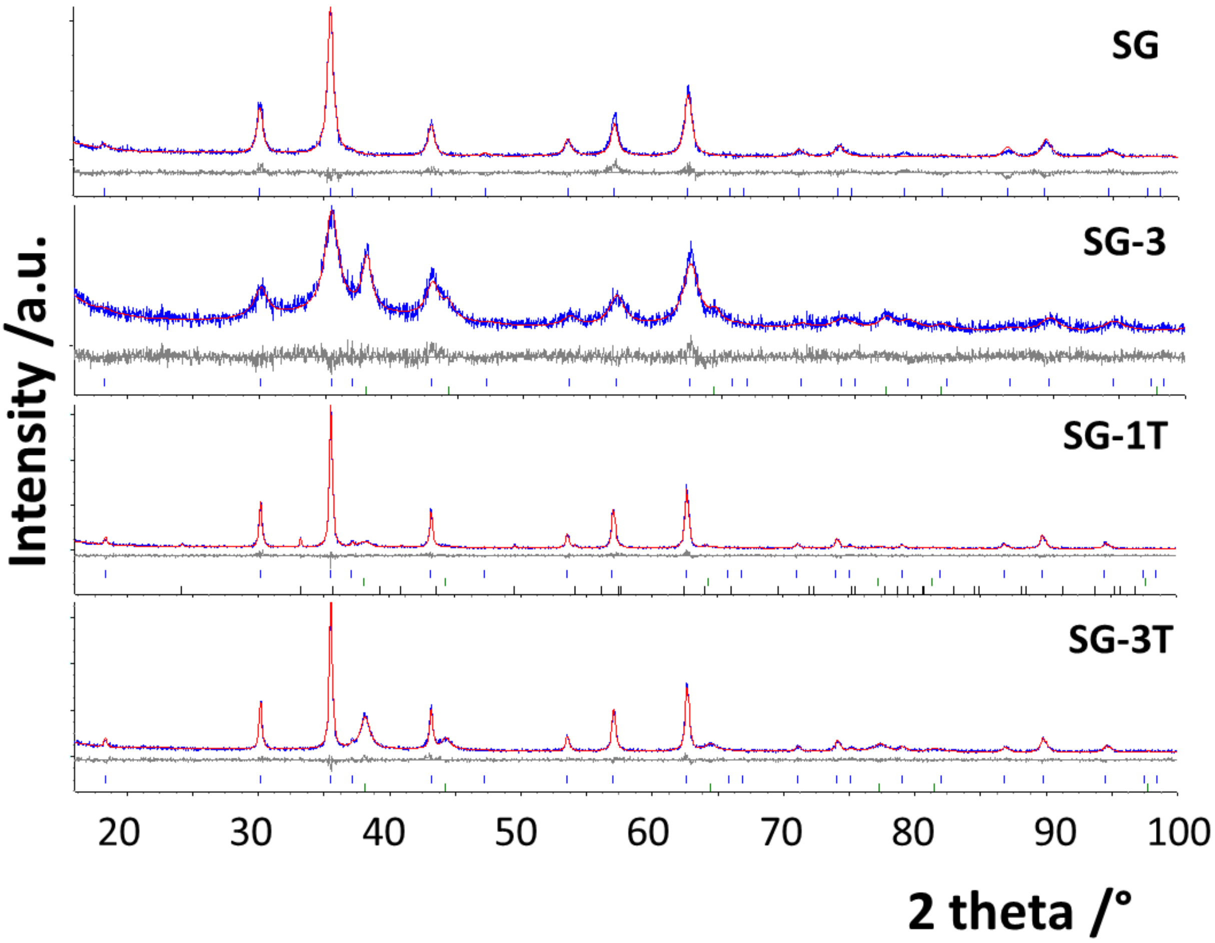

Figure S2. Rietveld refinements of the XRPD patterns of the SG samples indicated above the graph. The experimental curve (blue) is compared with the calculated one (red). On the bottom, the difference curve (grey) and the bars at the expected positions of the reflections for the different phases.

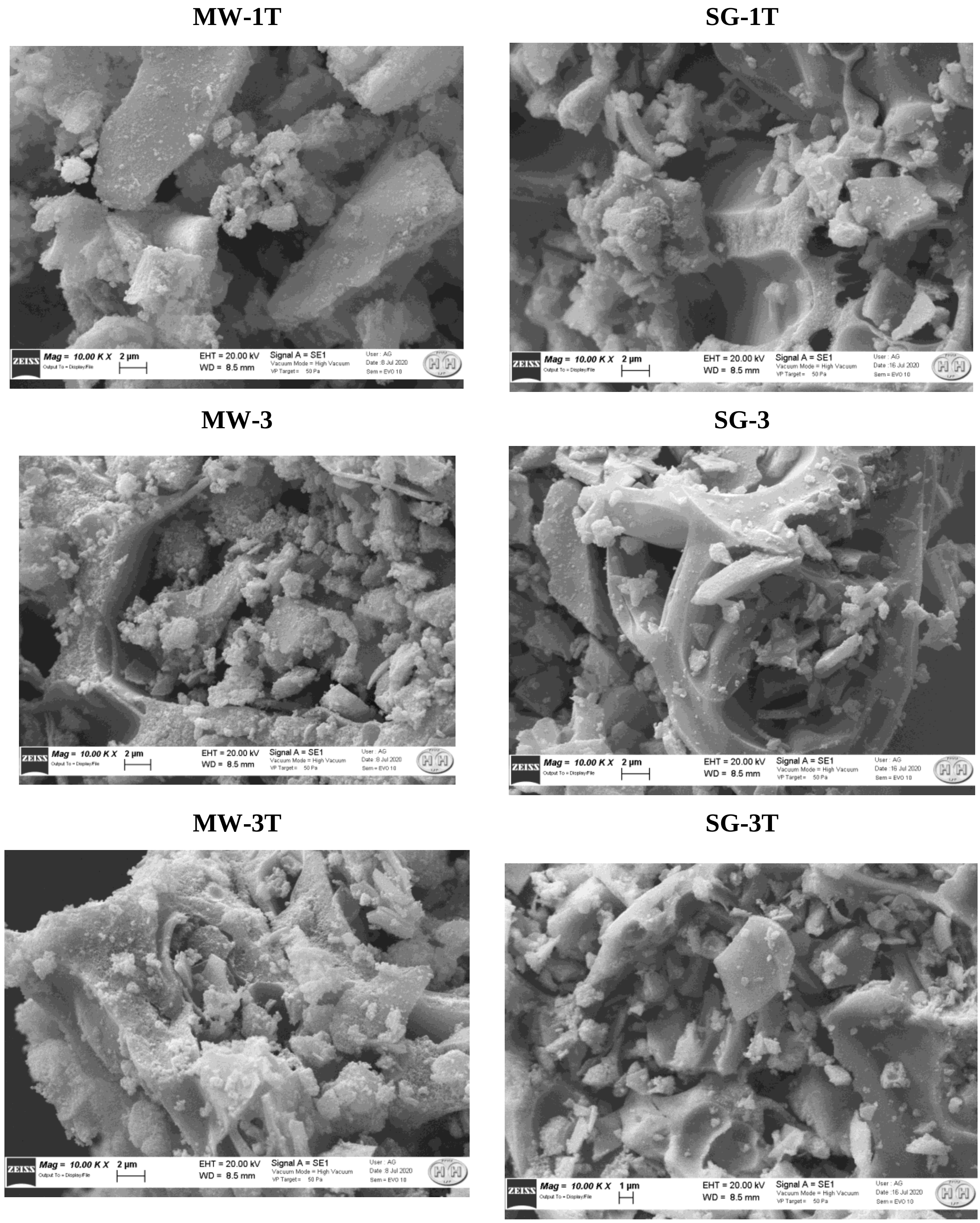

Figure S3. SEM images of the indicated samples, collected at the same magnification (10kx).

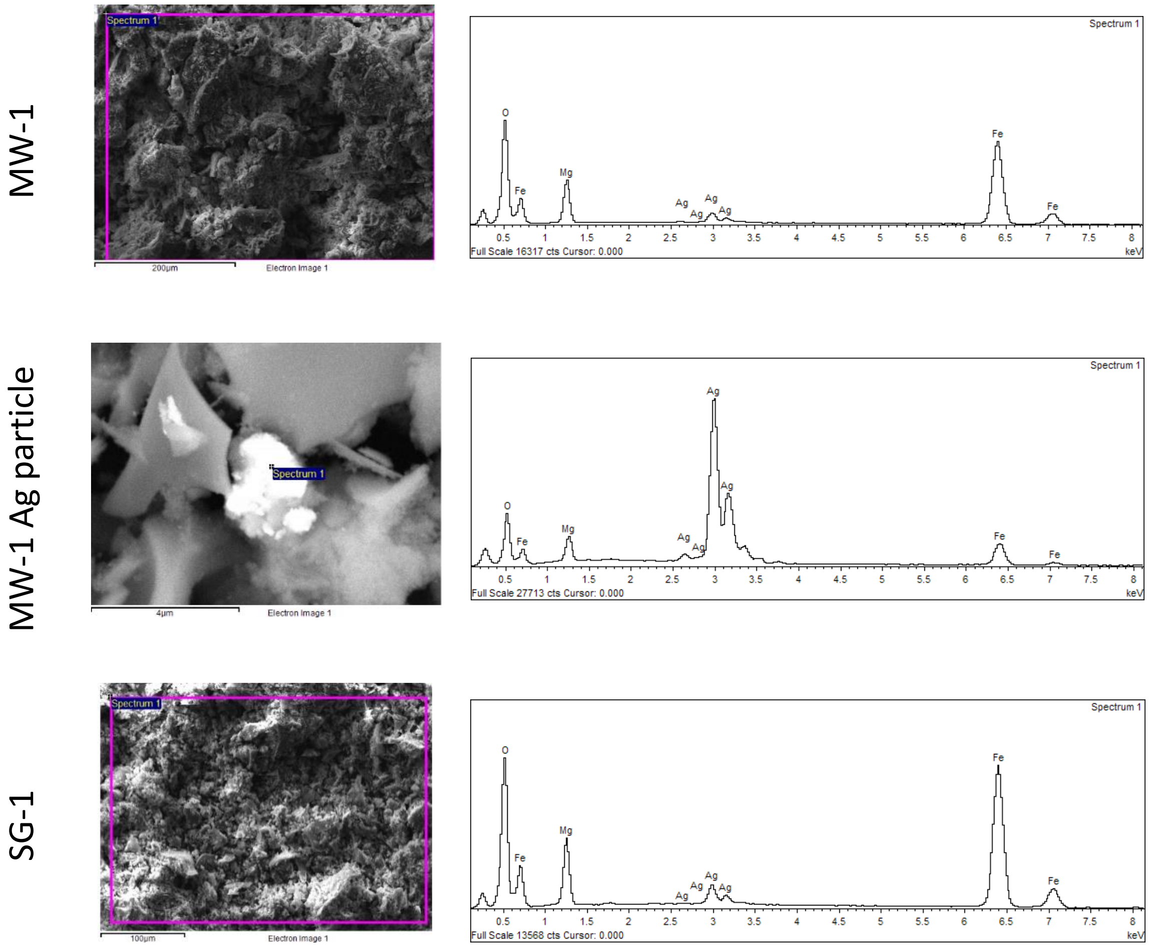

Figure S4. SEM images and the corresponding EDS spectra for the indicated samples.

Table S1. Atomic amount of the elements detected by EDS for pure and Ag 0.1 doped samples.

Sample	Mg at.%	Fe at.%	Ag at.%
MW	15.47	29.18	-
SG	14.51	28.10	-
MW-1	12.75	24.66	1.2
SG-1	12.82	26.79	1.5

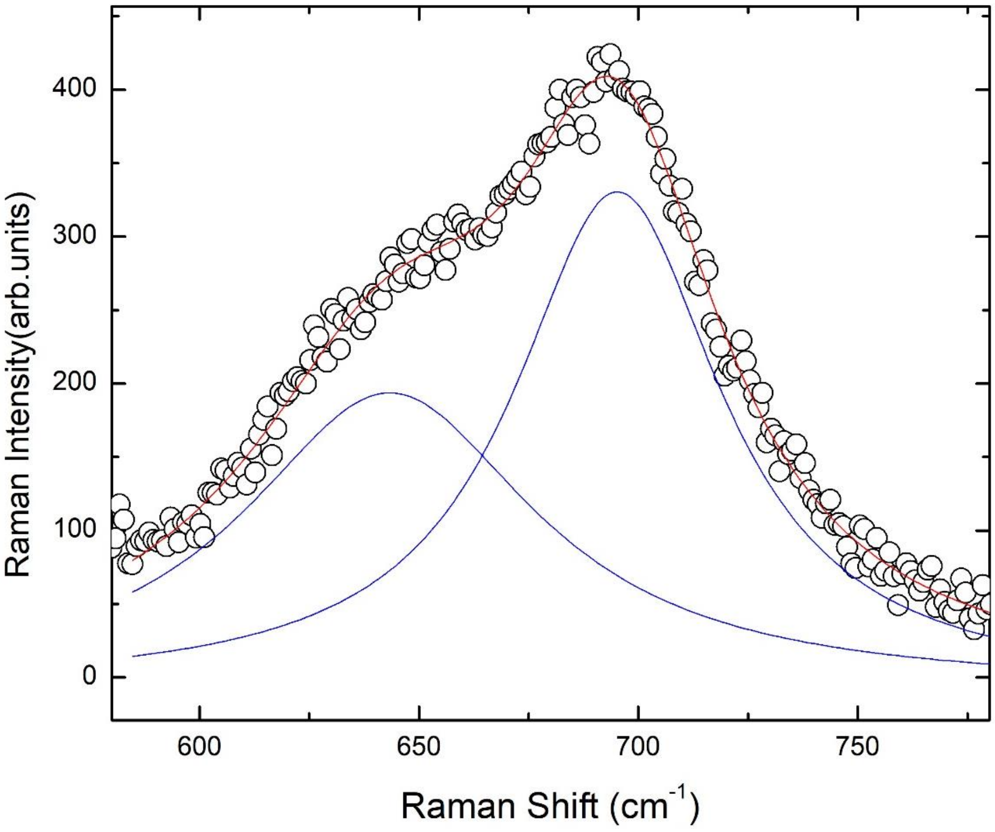

Figure S5. Example of the best fitting procedure using a sum of Lorentzian curves applied to MW sample Raman spectrum.
